# Meta-analysis of percutaneous radiofrequency ablation versus ethanol injection in hepatocellular carcinoma

**DOI:** 10.1186/1471-230X-9-31

**Published:** 2009-05-11

**Authors:** Carmen Bouza, Teresa López-Cuadrado, Raimundo Alcázar, Zuleika Saz-Parkinson, José María Amate

**Affiliations:** 1 Healthcare Technology Assessment Agency, Carlos III Health Institute, Madrid, Spain

## Abstract

**Background:**

Percutaneous radiofrequency ablation (RFA) has gained popularity in the treatment of hepatocellular carcinoma (HCC). However, its role versus other conventional minimally invasive therapies is still a matter of debate. The purpose of this work is to analyse the efficacy and safety of RFA versus that of ethanol injection (PEI), the percutaneous standard approach to treat nonsurgical HCC.

**Methods:**

Systematic review and meta-analysis of randomised or quasi-randomised controlled trials published up to August 2008 in PubMed, ISI Web of Science and The Cochrane Library. Overall survival, local recurrence rate and adverse effects were considered as primary outcomes. Studies were critically appraised and estimates of effect were calculated according to the random-effects model. Inconsistency across studies was evaluated using the I^2 ^statistic. Sensitivity analyses were conducted to explore statistical heterogeneity.

**Results:**

Six studies were eligible. The studies reported data on 396 patients treated by RFA and 391 treated by PEI. In general, subjects were in Child-Pugh class A (74%) and had unresectable HCC (mean size 2.5 cm). Mean follow-up was 25 ± 11 months. The survival rate showed a significant benefit for RFA over PEI at one, two, three and four years. The advantage in survival increased with time with Relative Risk values of: 1.28 (95%CI:1.12–1.45) and 1.24 (95%CI:1.05–1.48) for RFA versus PEI at 3- and 4-years respectively. Likewise, RFA achieved significantly lower rates of local recurrence (RR: 0.37, 95%CI: 0.23–0.59). The overall rate of adverse events was higher with RFA (RR:2.55, 95%CI: 1.8–3.6) yet no significant differences were found concerning major complications (RR:1.85, 95%CI: 0.68–5.01). There was not enough evidence supporting a better cost-effectiveness ratio for RFA compared to PEI.

**Conclusion:**

Available evidence from adequate quality controlled studies support the superiority of RFA versus PEI, in terms of better survival and local control of the disease, for the treatment of patients with relatively preserved liver function and early-stage non-surgical HCC. However, the higher rate of adverse events displayed is something that will have to be tested with appropriate weighting of the possible benefits in each individual case. Overall cost-effectiveness of RFA needs further evaluation.

## Background

Worldwide, hepatocellular carcinoma (HCC) constitutes an important problem for healthcare systems due to its high morbidity, mortality and progressive incidence [1]. HCC is the sixth leading tumour in the world [2], and it is estimated that its incidence will continue to rise in coming decades [3,4]. It is an aggressive tumour that usually develops in a cirrhotic liver with limited functional reserve, and without treatment registers a short survival after diagnosis [5]. Furthermore, unlike other tumours, HCC can be offered few possibilities of curative radical treatment [1]. Indeed, despite the fact that screening systems have improved the early diagnosis rate [6], the vast majority of patients are not susceptible to curative treatment when this tumour is detected [7].

Over the past few years, several methods for percutaneous tumour destruction have been developed [8,9]. Out of these, radiofrequency ablation (RFA) is the one that has attracted greatest interest and popularity and, presently, it is the most widely employed liver-directed treatment of early-stage non-surgical HCC [10,11]. Indeed, there has been a drastic shift from standard percutaneous treatments, such as Percutaneous Ethanol Injection (PEI), to RFA since the introduction of the latter in clinical practice [12].

Yet, to date, little is known of the efficacy and safety of percutaneous RFA versus that of other conventional minimally invasive loco-regional therapies [12-15], and the advantages of RFA versus PEI in terms of cardinal outcomes, such as survival, have not been demonstrated [1,13,14].

Accordingly, based on a systematic review and meta-analysis of the literature, this study sought to assess existing evidence about the efficacy and safety of percutaneous RFA versus that of PEI in the management of HCC.

## Methods

A review of the literature was conducted in PubMed, ISI Web of Science and The Cochrane Library from January 1990 to August 2008, using the following terms: "carcinoma, hepatocellular" [MeSH Terms], "radiofrequency catheter ablation" [MeSH Terms], "ethanol injection" [Text Word] and "controlled trial" [Publication Type]. Similarly, a manual search of the relevant references was made and experts were contacted in order to identify published studies [15]. Conversely, we did not attempt to contact companies producing RFA equipment.

### Inclusion criteria

Sackett's criteria [16], duly amended, were applied as follows: 1) population: randomised or quasi-randomised controlled studies conducted on more than 10 adults with formal diagnosis of HCC; 2) intervention: percutaneous RFA; 3) comparator: PEI; 4) results: studies were required to describe data related to at least one of the following primary variables of efficacy and safety, namely, overall survival, local recurrence rate and adverse effects. Other variables such as disease-free survival, complete tumour response and use of health-care resources were considered as secondary outcomes.

Since this study's aim was to identify existing evidence on the efficacy and safety of RFA versus PEI in the treatment of HCC, no limitations based on duration of follow-up period were established [15,17].

### Selection of studies

The selected studies were examined by two independent reviewers, with any disagreements being settled by discussion of the respective study data.

### Data extraction

Original data were extracted on a standard form that included: a) details of the study design, inclusion/exclusion criteria and duration of follow-up; b) information on the study population; c) information on the intervention and comparator; and lastly, d) information on the outcome measures of efficacy and safety.

### Analysis of methodological quality and scientific evidence

This was conducted in accordance with validated recommendations [18].

### Data analysis and synthesis of results

To obtain an overall measure of the efficacy and safety of RFA versus that of the comparator, standard meta-analytical techniques were used. Pooled effect was estimated using a random-effects model [19]. We analysed dichotomous variables using estimation of relative risk (RR) with a 95% confidence interval, and continuous variables using weighted mean difference (WMD) with a 95% confidence interval. The degree of inconsistency between studies was quantified using the I^2 ^statistic which describes the proportion of variance across studies not due to chance. I^2 ^<25% and I^2 ^>50% reflect small and large inconsistency, respectively [20]. Sensitivity analyses were conducted to explore statistical heterogeneity [21].

In accordance with some recent literature we have not used funnel plots to examine the possibility of publication bias, given the limitations and potential misleading results of these graphs [22].

All analyses were performed using the SE Stata 9 computer software package (StataCorp LP Texas USA 1984–2005). Results were deemed significant at a P-value < 0.05.

We used Visual RX Version 3 http://www.nntonline.net to calculate, where appropriate, the number needed to treat (NNT) or the number needed to harm (NNH).

## Results

**Figure 1:**
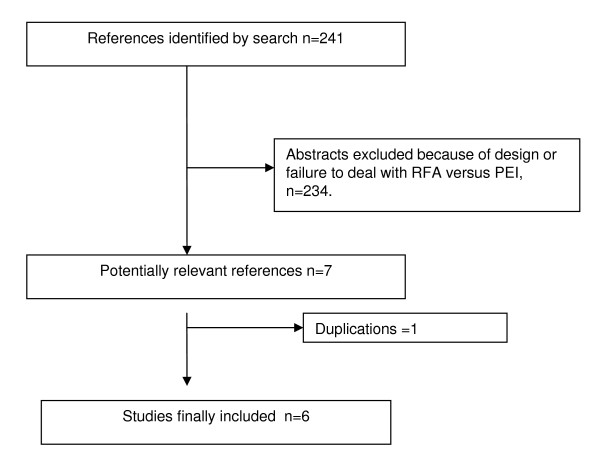
**Flow diagram for study selection and inclusion**.

As Figure 1 shows, the bibliographic search yielded 241 references. Hand searching of retrieved articles yielded no additional studies. After excluding references without an abstract, studies with a non percutaneous RFA technical approach, studies not reporting outcomes separately from data on patients with hepatic metastases, and redundancies arising from the use of several databases, a total of seven publications [23-29] were selected.

As Olschewski's [24] data are included within Lencioni et al.'s [25] (Table 1), a total of 6 studies, published from 1999 to 2008, finally met the inclusion criteria. The studies reported data on 396 patients treated by RFA and 391 treated by PEI. In general, subjects had unresectable HCC without vascular invasion and extrahepatic spread; mean size was 2.5 cm and 57% (619 of 1084 tumours) were single tumours. Most patients (74%) were in Child-Pugh class A (Table 1). Mean duration of follow-up was 25 ± 11 months. In every study serial assessments including clinical evaluations, analysis of tumour markers and imaging studies were usually performed during follow-up to assess the treatment response and to detect tumour recurrence. Shiina et al. [28] reported that, additionally, when local recurrence was suspected, a biopsy was performed.

**Table 1:** Percutaneous RFA *vs*. PEI. Summary of characteristics and quality of included studies.

Author/year of publication	Design	Inclusion criteria	RFA P, [T]	PEI P, [T]	Child-Pugh A/B/C	Mean follow-up (months/years)	Quality
**Livraghi^24^** **1999**	quasi-RCT	Cirrhosis/chronic hepatitis and HCC ≤ 3 cm.	42, [52]	44, [60]	RFA 39/3/0 PEI 38/6/0	10 m. (4–28)	2Diii

**Lencioni^26 ^2003** includes Olschewski^25^	RCT	Cirrhosis, single HCC ≤ 5 cm or 3 nodules ≥ 3 cm each. HCC at 1 cm hepatic hilum or gallbladder. No vascular invasion or extrahepatic metastasis. Child-Pugh: A or B. No previous treatment. No candidate for resection-transplantation.	52, [69]	50, [73]	RFA 45/7/0 PEI 35/15/0	RFA 22.9 ± 9.4 m. PEI 22.4 ± 8.6 m.	1iiA

**Lin^27^** **2004**	RCT	Cirrhosis, HCC 1–4 cm maximum. Child-Pugh A or B. No previous treatment. Tumour site >5 mm from the hilum or common bile duct.	52, [64]	52, [56]	RFA 41/11/0 PEI 39/12/0	RFA 24.5 ± 1.3 m. PEI 23.8 ± 10.4 m.	1iiA

**Lin^28^** **2005**	RCT	1–3 HCC ≤ 3 cm, a minimum of 1 cm from the hilum and gallbladder, no vascular invasion or extrahepatic metastasis. Child-Pugh A or B cirrhosis. Initial treatment.	62, [78]	62, [76]	RFA 46/16/0 PEI 47/15/0	RFA 28 ± 12 m. PEI 26 ± 13 m.	1iiA

**Shiina^29^** **2005**	RCT	Unresectable HCC or patient's refusal of surgery. ≤ 3 lesions ≤ 3 cm. Child-Pugh A or B. No extrahepatic metastasis or vascular invasion. No previous or simultaneous malignancy.	118, [187]	114, [192]	RFA 85/33/0 PEI 85/29/0	RFA 0.6–4.3 y. PEI 0.1–4.2 y.	1iiA

**Brunello^30^** **2008**	RCT	Cirrhotic patients in Child-Pugh A or B with 1–3 HCC nodes ≤ 3 cm. Tumour site ≥ 1 cm from the hilum, gallbladder, colon or stomach. No venous invasion, no metastatic disease. Patients no suitable for resection or liver transplantation.	70, [89]	69, [88]	RFA 39/31/0 PEI 39/30/0	RFA 26.1 m. PEI 25.3 m.	1iiA

RCT: randomised controlled trial. RFA: Percutaneous radiofrequency ablation. PEI: Percutaneous ethanol injection; P: patients; T: tumours.

Quality of studies: all based on reference 19.

As Table 2 shows, there were no significant differences between groups as regards Child-Pugh grade of liver dysfunction, tumour size, number of single tumours and duration of follow-up.

**Table 2:** P-RFA vs. PEI: Results of meta-analysis

Variables	No. of studies furnishing data	Results		RR/WMD (95% CI) P-value	I^2^
		RFA	PEI		
**Baseline characteristics:**					
Child-Pugh grade of liver dysfunction A B	6^24,26–30^ 6^24,26–30^	71% 24%	70% 26%	1.05 (0.97, 1.13), 0.44 0.92 (0.74, 1.15), 0.43	0% 0%
Tumour size, cm (mean ± SEM)	4^26–28,30^	2.62 ± 0.33	2.47 ± 0.35	0.13 (0.01, 0.25),0.03	0%
Single nodule	6^24,26–30^	59%	55%	1.04 (0.94, 1.50), 0.44	17%
Follow-up duration, months (mean ± SEM)	3^26–28^	25 ± 6.2	23.6 ± 5.6	0.97 (-1.32, 3.3), 0.4	0%
**Efficacy:**					
Survival 1 year 2 years 3 years 4 years	5^26–30^ 5^26–30^ 4^27–30^ 2^29,30^	96% 86% 73% 62%	91% 75% 58% 51%	1.04 (1.007, 1.08), 0.02 1.13 (1.06, 1.20), <0.001 1.28 (1.12, 1.45), <0.001 1.24 (1.05,1.48), <0.001	0% 0% 12.6% 0%
Local recurrence	4^26–29^	7%	22%	0.37 (0.23, 0.59), 0.000	0%
Disease-free survival 1 year 2 years 3 years	3^26–28^ 3^26–28^ 2^27,28^	80% 61% 40%	70% 42% 19%	1.13 (1, 1.28), 0.04 1.31 (1.06, 1.61), 0.013 2.1 (1.35, 3.23), 0.001	0% 0% 0%
Tumour complete response	4^24,26–28^	93.5%	84.5%	1.10 (1.04, 1.17), 0.01	0%
Remote intra-hepatic recurrence	5^26–30^	43%	45%	0.97 (0.82, 1.11), 0.56	0%
**Safety:**					
Total complications	6^24,26–30^	19.2%	10.5%	2.55 (1.8, 3.65), <0.001	0%
Major complications	4^24,28–30^	4.1%	2.7%	1.85 (0.68, 5.01); 0.22	0%

RR: Relative risk. WMD: Weighted mean difference. All based on random effects meta-analysis.

### A) Clinical efficacy

Selected studies report data on overall survival over different time intervals and only in the first 4 years of treatment (Table 1). At 1 year, meta-analysis shows a survival rate of 96% (95%CI: 92%–98%) in the RFA and of 91% in the PEI treated-group with differences being small but statistically significant in favour of RFA (Table 2). Pooled analyses of the five studies that furnished data on survival at 2 years found an overall survival rate of 86% (95%CI:80%–90%) in the RFA treated-group (n = 354). Meta-analysis shows, with large consistency across trials, a significant difference in favour of RFA (Table 2) being the calculated NNT of 11 (95%CI:7–23).

**Figure 2:**
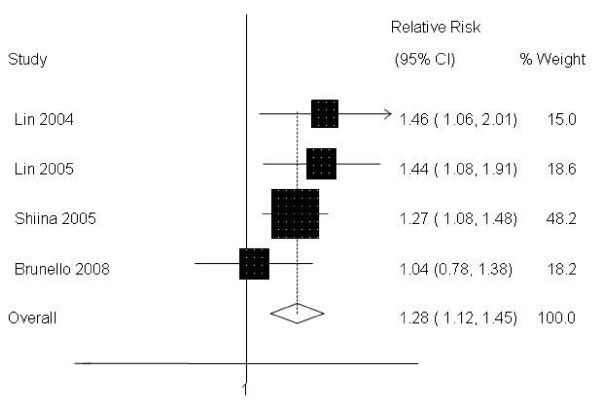
**RFA vs. PEI: Results of the meta-analysis on overall survival at 3 years**. CI: Confidence interval. All based on a random-effects meta-analysis.

At 3 years, the pooled analysis of the 4 studies furnishing data shows that 74% (95%CI: 66%–86%) of RFA treated-patients (n = 302) survived. Furthermore, this analysis shows, also with a high degree of consistency, that RFA achieved a significantly higher survival rate than PEI (Table 2, Figure 2) with a calculated NNT of 7 (95%CI:4–15).

Data from the two studies analysing this variable at 4 years indicate 62% survival rate (95%CI: 54%–75%) in the RFA-treated group. Joint analysis also shows a statistically significant improvement in survival in this group compared to PEI (Table 2). The calculated NNT here is 9 (95%CI: 5–40).

**Figure 3:**
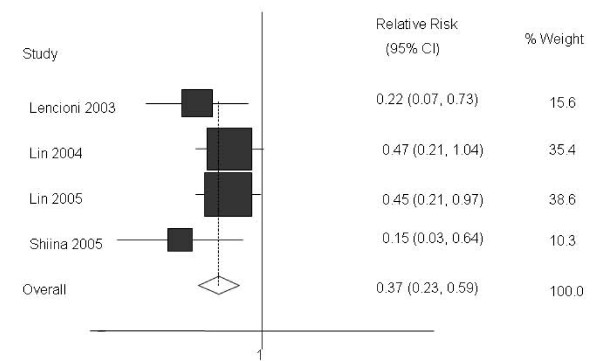
**RFA vs. PEI: Results of the meta-analysis on local recurrence rate**. CI: Confidence interval. All based on a random-effects meta-analysis.

Furthermore, pooled analysis of studies furnishing data found that, at the end of the follow-up period, the rates of local tumour recurrence were 7% (95%CI: 4%–10%) and 22% (95%CI: 17%–27%) in the RFA and PEI treated-groups respectively. These differences were found to be statistically significant (Figure 3, Table 2), with a calculated NNT of 9 (95%CI: 6–25).

Concerning secondary outcomes, as Table 2 shows, our results indicate that one, two and three year disease-free survival rates were significantly higher in the RFA group. Likewise RFA was accompanied by a significantly higher rate of radiological complete necrosis. Conversely, no statistical differences were found as regards remote intra-hepatic recurrence outside the treated field.

### B) Safety

Table 3 lists the complications reported in individual studies. Overall, complications were described in 19% of patients (95% CI: 15%–23%) treated by RFA and in 10.5% (95% CI:7–13.5%) of those treated by PEI, with the difference being statistically significant and favourable to PEI (Table 2) and an estimated NNH of 7 (95%CI:4–13) with RFA.

**Table 3:** RFA vs. PEI. Summary of complications described in individual studies

Type	RFA (number of cases)	PEI (number of cases)
Severe pain	27	14
Fever	10	5
Haemothorax	4	
Pneumothorax	1	
Pleural effusion	7	
Gastric perforation	1	
Cholecystitis	1	
Intraperitoneal bleeding	2	1
Haemobilia	1	
Hepatic infarction	1	
Subcapsular haematoma	5	1
Transitory icterus	1	
Ascitis	1	2
Acute cholangitis		1
Hepatic abscess		1
Portal vein thrombosis	2	9
Biliary stricture	1	
Hypertransaminaemia	3	1
Thermal burn to colon	1	
Cutaneous burn	1	2
A-V shunt	3	
Neoplastic cell seeding	3	1
Psychotic reaction		1
Vagal reaction		1
Procedure-related death		1

Only four studies report on complications defined as being major by the respective authors. The rate of major complications in the RFA treated-patients was 4.1% (95%CI: 1.8%–6.4%) and include: haemotorax requiring thoracostomy drainage, gastric bleed, haemoperitoneum, transitory icterus, liver infarction, cutaneous burn and tumoral cell seeding. In the PEI-group, 2.7% (95%CI: 0.4%–5.1%) of patients had major complications including: liver abscess, haemoperitoneum, tumoral cell seeding and one procedure-related death. Pooled analysis of these major complications, showed that, despite there being a trend towards a greater number in the RFA group, this difference failed to reach statistical significance (Table 2).

### C) Use of resources

Joint analysis of four studies [25-28] indicates that the number of sessions per tumour treated was significantly lower in the case of RFA (WMD: -4.26, (-4.8, -3.7), <0.001; I^2^:88%). When potential reasons for heterogeneity were explored, the studies varied in terms of both clinical variables and methodological quality. However, a range of sensitivity analysis showed no appreciable differences between the pooled-effect sizes obtained.

Three studies [26-28] furnished combinable data on mean hospital stay. In two of these, hospital stay was longer in the RFA-treated group, whilst Shiina et al. report data on very prolonged stays in the PEI-treated group, without citing the causes. Although combined analysis showed no significant differences [WMD: -2.91 (-7.91, 2.08), P = 0.2], inconsistency across studies was very pronounced (I^2^:99%), rendering interpretation of results difficult.

Only Brunello et al. [29] conducted a formal evaluation of hospital costs. These authors found that the mean direct medical costs were 4097ℇ for patients in the PEI group and 6540ℇ for those in the RFA group (p < 0.001). In addition, they estimated an incremental health-care cost of 8286ℇ (95%CI: 2742–20 917 ℇ) for each additional patient successfully treated by RFA.

## Discussion

The results of this meta-analysis indicate that RFA is superior to PEI in terms of overall survival and lower local recurrence rates for patients with Child-Pugh class A or B cirrhosis and an early nonsurgical HCC. However, the higher rate of adverse events displayed is something that will have to be tested with appropriate weighting of the possible benefits in each individual case.

While according to recent guidelines PEI should be considered the standard technique for percutaneous treatment of HCC in patients with cirrhosis [30], RFA has emerged as a real competitor to PEI in clinical grounds and is currently used as the primary ablative modality at most institutions [31,32]. However, to date, the survival benefit of RFA versus that of PEI for HCC is controversial [1,13,14,31].

In this study, a significant benefit in RFA vs. PEI in overall survival was observed. Interestingly enough, though individual studies provide data only in the first 4 years of treatment, the advantage in survival increases with time. Indeed at 3 years there is a 28% higher survival rate for the RFA-treated group with a calculated NNT of 7. From a clinical point of view these findings seem to be highly significant, and although the number of studies furnishing combinable data is limited, the absence of patent differences between groups with regards to Child-Pugh grade of liver dysfunction, tumour size or number of single tumours, highlights the possible benefits of RFA itself. Furthermore it is important to emphasize the high degree of consistency found across studies and their methodological quality, something that increases the strength of these results. In addition, the rates of survival found in the PEI group, while somewhat lower, are comparable to that reported in prior studies conducted in patients with HCC of similar characteristics [33,34].

The improvement observed regarding survival can be explained by the fact that, as this study confirms, RFA is superior to PEI in achieving local control of the disease as demonstrated by its greater disease-free survival rates, complete radiological tumour response, and its significantly lower local recurrence rates [31,35-38]. Concerning this cardinal variable, our data show, with enormous consistency across the combinable studies, that RFA has a 63% lesser risk of local recurrence than PEI with a calculated NNT of 9 (95%CI: 6–25). Conversely, and as expected because it is not influenced by local treatment, no significant differences were found concerning remote hepatic recurrence rates outside the treated field [38].

Insofar as safety is concerned, though data are limited, available evidence indicate that, whereas there are no significant differences between the two techniques in the case of major complications, RFA displays an overall complication rate which is significantly higher than that observed with PEI, with a calculated NNH of about 7.

The overall rate of complications was 19% in the RFA treated-group, which is clearly higher than the 7% previously reported for this percutaneous approach [39]. On the other hand, we should point out that the rate of major complications found in this study (4%) is rather comparable to recently published data [40]. While these rates do not translate into an increase in mortality they have to be taken into account, especially because this procedure has rapidly evolved into the most popular percutaneous therapy for HCC in clinical settings. Here, we must point out that some of the complications observed could be due to the effect of the learning curve [41] and professionals' differing degree of experience [42] in RFA.

Lastly, RFA seems superior to PEI in terms of some health-resource-related variables, such as number of sessions per tumour treated and mean hospital stay. In contrast, in the cost-effectiveness analysis made by Brunello et al. [29], RFA was found to be more expensive than PEI with mean direct costs showing a significant positive difference not balanced by a better impact on the overall survival rate. Consequently, to date there is no evidence supporting a better cost-effectiveness ratio for RFA from a social perspective. In addition, it is important to recognise the difficulties in comparing the length of hospital stay and the costs of RFA versus PEI among studies of diverse setting due, in part, to the difference in resource use and patterns of care between different countries. These differences also hinder direct comparisons and extrapolation of the results to different communities.

### Potential report limitations

It has to be recognised that publication bias is possible and that, by not including unpublished studies, the efficacy and safety of RFA versus PEI may not have been fully adequately estimated. Nevertheless, we feel that any such bias would necessarily have been minimised by the scope of and systematic strategy used in the search of the literature and we are confident that the majority of the research conducted in this field was successfully identified [15,43]. However, in line with prior reports, we decided not to include unpublished data from industry given both the difficulties encountered in obtaining this information and the recognition that the use of these data may not necessarily reduce the bias in meta-analysis [43,44].

We should, moreover, stress the fact that, in this study, a number of accredited methods were used to reduce possible biases (extensive search of the literature; duplicate data-extraction; use of explicit criteria for both methodological assessment and analysis; and use of a random-effects model for effect estimation). Additionally, cardinal clinical results as overall survival were analysed [45].

Lastly, this study highlights the presence of several limitations in the papers located in the literature among which the cursory description of results and the absence of stratification according to recognised prognostic parameters with regards to both tumour size and hepatic functional reserve, reduce the possibility of the applications and clinical limitations of RFA being identified more accurately. In this regard, it has to be recognised that although the efficacy of RFA is known to be size dependent [36], no subgroup analysis based on tumour size could be carried out given the absence of adequate information on this respect in the individual studies. Similarly, there is an evident lack of long-term data on patient survival. Most of the studies had follow-up periods of approximately 2–3 years and there are no consistent data on longer follow-ups. There is no doubt that this is a limitation which will be overcome in the future, rendering it possible for patient prognoses to be correctly evaluated.

## Conclusion

Despite limitations, available evidence from adequate quality controlled studies support the superiority of RFA versus PEI in terms of better survival and local control of the disease for the treatment of patients with relatively preserved liver function and early-stage non-surgical HCC. However, the higher rate of adverse events displayed is something that will have to be tested with appropriate weighting of the possible benefits in each individual case. Overall cost-effectiveness of RFA needs further evaluation.

## Competing interests

The authors declare that they have no competing interests.

## Authors' contributions

CB participated in the design and coordination of the study, carried out the critical appraisal of studies and wrote the manuscript. RA, ZSP and TLC developed the literature search, carried out the extraction of data, assisted in the critical appraisal of included studies and assisted in writing up. CB and TLC carried out the statistical analysis of studies. JMA coordinated the project (SEC 2001-0138) and assisted in writing up. All authors read and approved the final manuscript.

## Pre-publication history

The pre-publication history for this paper can be accessed here:

http://www.biomedcentral.com/1471-230X/9/31/prepub
